# Discharged. Not Where to Lay His Head

**Published:** 1886-11-06

**Authors:** George Fleming


### Discharged.

### "NOT WHERE TO LAY HIS HEAD."

By George Fleming.

VII.

As Lemon entered the dimly lit passage, where the smell of the mid-day mutton still clung to the damp walls, the landlady's youngest boy, Tommy, came running out to meet him, to say that the other lodgers were all gone out, and mother says we're a' having supper in the back parlour if you care to come in. And the solid figure of the good woman of the house, placidly conscious of vast expanses of best black silk, appeared in the narrow doorway to confirm the invitation.

"For, as I said to Maria, them as has been ill is ever given to moping," she said genially, making room for Lemon to pass. "It's all very well for them like Amelia Jane there, as is too young yet to know what it is to get a sinkin' in one's insides, but them as has been through it knows what sinkin' is. And so I told her only just now as I heard you coming in. For there is that young man upstairs, says I, who hasn't to my knowledge so much as picked at a bit of hot victual this livelong day. And here's ourselves and friends, sitting down as comfortable as comfortable, to a meal that hasn't met with no scrimping. I'll say that much for it, though I cooked it myself."

"Which anyone else would be glad and ready to say for you, Sarah," observed the thin lady of a certain age, who was seated on the edge of the horsehair sofa on the opposite side of the room. "Though I've ever been brought up in the belief that praise to the face is open disgrace," she added slowly, fixing her gaze upon Lemon's flushed cheeks and bright eyes in a manner which made the remark sound painfully personal.

The rest of the company consisted of the disdainful young lady with the ringlets, a young man in attendance upon her, who wore a blue necktie, and carried a prayer-book and a hymn-book in either pocket, from whence they were constantly falling to the floor, thereby giving rise to considerable badinage, and much tossing of the aforesaid ringlets. A large, pale man who never spoke, but who would have described himself as the master of the house, and the small boy, Tommy, completed the gathering.

The hot spirit which Lemon had swallowed had not yet lost its potency; and although he was forced to decline a good deal of Mrs. Pink's pressing hospitality, so causing that worthy woman to protest with much vehemence "that no good could come to a young man who slighted his victuals, and that she *would* say, whoever might choose to contradick it," he yet developed social talents on that occasion which contributed not a little to the hilarity of the evening. It is true that, despite all the time bestowed upon his education, Spot could not be trusted to beg without a succession of proddings and pokings, which detracted sadly from the general effect; and in the matter of expressing his political opinions, too, he gave three barks for Mr. Gladstone, or for Lord Salisbury, with an impartiality which was discouraging even in a mongrel. Still, as Mr. Pink observed solemnly, "there's other people puzzled to say which, beside dogs." And Lemon made himself universally agreeable. His weak, affectionate nature basked in this atmosphere of good-natured approval. When Miss Pratt objected to all tricks done with playing-cards as un-Sabbatical and not to be tolerated, and made trenchant allusions to those who waste their substance in strong drink and riotous living, he agreed with her instantly, and at once volunteered to run up to his room and fetch down the frame he had been carving for his pledge-card.

He was away a good deal longer than might have been expected; a delay which he explained by remarking that going up stairs at that pace, or something, had given him a nasty pain in his side. Mrs. Pink recommended essence of peppermint as of sovereign virtue as a remedy in such cases; and the ingenious little frame was passed from hand to hand with much admiring comments.

When it came back to Lemon he, too, smiled upon it approvingly. "That ain't nothing special; as work, I mean. It's only done with a penknife. But on Tuesday, when I get the stuff, I'll finish it up with a bit o' gilding. I made it," he said—and then he thought of Mary—"I just made it for a bit of remembrance for a friend."

VIII.

It was getting on for eleven o'clock that same night; Mrs. Pink was just dropping off into her first comfortable sleep, when the sound of stealthy footsteps in the passage, and what seemed like the fumbling of an unaccustomed hand about the fastenings of the front door, roused her into a state of acute wakefulness. There were other lodgers in the house besides those for whom she was responsible, but the good woman sat up in bed and listened with an anxiety which she afterwards characterised as "profitical."

"Pink! Pink! Lord bless the man! You might be robbed an' murdered an' have your throat cut in your own bed, an' never find it out till you was called next morning. Pink! wake up, I say. I tell you when I heard that door shut I heard the new lodger's dog bark as plain as plain!"

"Well? can't he bark without me and you to help 'im?"

"But, Pink," the good woman said anxiously, "you never *will* open your eyes to what's goin' on straight before you. And Maria Pratt de- clares that when that young man came in this evening she began to smell spirits on the spot."

"Maria Pratt is a skinny, long-tongued old maid," growled Pink, who was sleepy, and cherished all the contempt of the middle-aged unattractive male being for the contemporary woman.

But good Mrs. Pink lay awake a long time listening to the rain beating against the window. "Dear, dear!" the kindly woman sighed to herself, "to think of all the poor souls in want of a friend to-night!" Her thoughts wandered from reflections about the cut of Amelia Jane's new purple basque, to a remembrance of Lemon's haggard cheeks. "If he had only some place to go to; some one to look after him and keep him steady, just for the first week after comin' out o' hospital!" she said to herself, sighing again. For Mrs. Pink, too, had had her experiences,—in the dark days before Pink subsided into his present condition of grim but unresisting respectability;—and perhaps it was only natural that the woman who had endured those things should remember them longer than the man through whom they had come.

At the same hour, and not so very far away in point of space, a very different scene was being enacted.

For, even on this wet Sunday evening, a few idlers,—the nucleus of what would have been an animated crowd under more propitious circumstances,—were gathered about the door of one of the Knightsbridge public-houses, from which two policemen were half carrying, half leading away a drunken man. The light from the row of gas-burners fell full upon his white face, his torn shirt, and the dishonoured head from which the hat had fallen; it glistened, too, upon the policemen's metal helmets and dripping mackintosh coats.

"'E's got '*is* qui-e-tus. Been tryin' some of the Very Mild and Pleasant!" one humourist cried out in playful allusion to the placard conspicuously swinging above the door.

The laugh excited by this remark prompted another spectator to inquire politely if any gentleman present could explain how it was that the small dawg 'ad come out in spots? and it may have been the hoarse roar which greeted this question, or it may have been the sharp touch of the pure outer air, which made Lemon suddenly lift up his head and gaze about him with stupid, blood-shot eyes.

"It's all right, mates," he said. "There's work a comin'—Tuesday."

He stumbled heavily forward, and would have fallen prone in the mud but for the quick hand of the nearest policeman.

IX.

Some little time before dawn a policeman carrying a lighted lantern, and with a face even graver than was exacted by the proprieties of the profession, tapped at the door of the Inspector's private room.

"That man X 31 brought in to-night, sir;—drunk and disorderly from Knightsbridge;—something very queer about him, I'm afraid, sir. Broken blood-vessel, or something of the kind, I'm afraid, sir."

"Hallo," said the Inspector, jumping up promptly to his feet, "that's bad." He began searching under the sofa for his boots. "Where have you put him?"

"No. 3 cell, sir. The doctor's been with him this last hour, but he's been bleeding dreadful. Thought perhaps you would just come and have a look at him yourself, sir."

The two men went out together into the passage. There was a savage sound of woman's laughter and a vigorous beating of fists upon the door of No. 4; but 3 cell was quiet enough in all conscience. The door had been left open for air, and the doctor, who could be seen bending over a recumbent figure, looked up as the Inspector entered.

He stood for a moment in silence, looking down at the blood-stained bed. "Going fast, eh?"

"Hæmorrhage of the lungs.—No; I should say there had been no striking. He was evidently only just convalescent; been well nursed, too. I should say that even a moderate amount of drink might have brought on the relapse."

The four men present were again silenced for a little. Then, "Got his name, have you?" the Inspector asked, looking across at 31 X.

"No, sir. When we took him he was much too far gone to give it, sir."

"H—m. Well. You'd better go round to the public, and see if they know anything about him in the morning. There'll be the inquest and——Hallo! Who let that dog in here?"

He pointed to poor Spot, who was curled up in as small a space as possible in the darkest corner beneath the bench. The policemen both looked foolish; and one of them said apologetically, "Well, sir, he followed us in somehow; slipped in between our legs when we weren't looking. And he's been sitting there ever since, only whining to himself like a baby."

The doctor had been feeling Lemon's pulse. He laid his patient's arm down again, quite gently, on the blanket. "I'll go across with you, Mr. Inspector, if you will just wait while I wash my hands. There won't be any change now. He may be an hour or two sinking. But you can call me if I'm wanted again."

Perhaps half-an-hour later, the man who was seated nearest to Lemon saw an indescribable change—a movement of the muscles, like the ripple under water—pass over his blood-stained face. He opened one of his eyes. "There warn't no other place," he said faintly, but quite distinctly.

The policemen looked at each other, and one of them got up and leaned over the bed.

"What place, my man? Do you want anything?"

"It was all—Dick's money—I did not mean—to steal it," the sick man muttered restlessly. "There wasn't—no—other—place."

The sound of his voice had brought poor, loving Spot shivering and crawling out of his place of refuge. He pressed up against the side of the bed now, and whined, licking his master's hand. It seemed impossible that Lemon should recognise the touch, but yet, and for the last time, his sunken eyes brightened faintly. A pale smile, the ghost of his old joy in life, passed like a reflection across his grey lips. "There's the work — comin'— all right on Tuesday," he murmured vaguely. Then, after a minute: "Bed 71—workhouse infirmary — that's the ticket."

Presently dawn appeared; turning to a gleaming square the dirty, grated window. Then the day broke over the great city in which four millions of human beings are for ever going about their business or their pleasure—every man with his own experience to make; every man with his own experience to answer for—unconscious of each other in life as in death.

